# A systematic review of nutraceutical interventions for mitochondrial dysfunctions in myalgic encephalomyelitis/chronic fatigue syndrome

**DOI:** 10.1186/s12967-021-02742-4

**Published:** 2021-02-17

**Authors:** Rebekah Maksoud, Cassandra Balinas, Sean Holden, Hélène Cabanas, Donald Staines, Sonya Marshall-Gradisnik

**Affiliations:** 1 National Centre for Neuroimmunology and Emerging Diseases (NCNED), Menzies Health Institute Queensland, Griffith University, Gold Coast, Australia; 2 Consortium Health International for Myalgic Encephalomyelitis, Griffith University, Gold Coast, Australia; 3 School of Medicine, Griffith University, Gold Coast, Australia

**Keywords:** Myalgic encephalomyelitis, Chronic fatigue syndrome, Mitochondria, Nutraceuticals, Intervention

## Abstract

**Background:**

Myalgic Encephalomyelitis/Chronic Fatigue Syndrome (ME/CFS) is a debilitating illness, characterised by persistent fatigue that is unrelieved by rest, in combination with a range of other disabling symptoms. There is no diagnostic test nor targeted treatment available for this illness. The pathomechanism also remains unclear. Mitochondrial dysfunctions have been considered a possible underlying pathology based on reported differences including structural and functional changes in ME/CFS patients compared to healthy controls. Due to the potential role that mitochondria may play in ME/CFS, mitochondrial-targeting nutraceutical interventions have been used to potentially assist in improving patient outcomes such as fatigue. The aim of this systematic review is to appraise literature assessing these nutraceuticals as a possible intervention for treating ME/CFS.

**Methods:**

A systematic search of Pubmed, Embase, Medline (EBSCO host) and Web of Science (via Clarivate Analytics) for journal articles published between January 1995 and 10th November 2020 was conducted. Articles assessing nutraceutical interventions and ME/CFS patient outcomes were retrieved. Using specific inclusion and exclusion criteria, the list of articles was further refined. Quality was measured using the Rosendal scale.

**Results:**

Nine intervention studies were included in this review. The studies investigated patient symptom severity changes such as altered fatigue levels in response to mitochondrial-targeting nutraceuticals. Improvements in fatigue levels were observed in six of the nine studies. Secondary outcomes assessed include biochemical, psychological, and quality of life parameters.

**Conclusion:**

There is insufficient evidence on the effectiveness of mitochondria- targeting nutraceuticals in ME/CFS patients. Future well-designed studies are required to elucidate both the involvement of mitochondria in the pathomechanism of ME/CFS and the effect of mitochondrial-modifying agents on illness severity.

## Background

Myalgic Encephalomyelitis/Chronic Fatigue Syndrome (ME/CFS) is a multifaceted illness characterised by persistent fatigue made worse by physical exertion. This fatigue is unrelieved by rest and is accompanied by a combination of symptoms such as immune, cardiovascular, endocrine, and neurological disruption [1–4]. Presentation and severity of symptoms varies between patients and leads to decline in overall quality of life, social, occupational, and personal activity where some patients are even bedbound [5]. There is no diagnostic test, thus classification of ME/CFS patients is dependent on case criteria when all other potential clinical explanations are excluded [1–4].

There are four main criteria used both in research and clinical practice to aid diagnosis of ME/CFS: the 1994 Fukuda Criteria (FC), 2003 Canadian Consensus Criteria (CCC), 2011 International Consensus Criteria (ICC), and 2015 Institute of Medicine Criteria (IOMC) [1–4]. FC has been criticised as being too broad, with considerable overlap with other conditions suggesting this is a barrier for appropriate diagnosis, it however, remains the most frequently used case definition [1–4]. The CCC, ICC and IOMC include more ME/CFS specific symptoms such as post- exertional malaise [1, 3, 4]. A common feature in all these definitions is persistent fatigue unrelieved by rest being listed as a cardinal symptom [1–4]. As fatigue is a key diagnostic symptom for ME/CFS, it has been suggested that energy metabolism may be disrupted in ME/CFS pathomechanisms.

Mitochondria are membrane bound organelles that have diverse physiological roles including energy production and cell cycle control. Mitochondria consist of an inner and outer membrane that separate three core regions: the aqueous regions, the intermembrane space, and the matrix. Within the inner region also resides the electron transport chain (ETC). The ETC comprises of five multi-subunit enzyme complexes (complex I through V) and two electron carriers: coenzyme Q_10_ (CoQ_10_) and cytochrome c which are necessary for oxidative phosphorylation leading to production of adenosine triphosphate (ATP)[6]. ATP is the energy currency used by the body to function. In addition to energy production, mitochondria are also key moderators of intracellular calcium (ca^2+^) homeostasis as well as immune regulatory pathways [7, 8]. Due to their physiological versatility, mitochondria have been implicated in numerous pathological conditions including metabolic and age-related disorders as well as ME/CFS [6, 9–27].

A recent systematic review investigated mitochondrial dysfunction in ME/CFS patients compared with healthy controls (HC)[28]. Although disruptions to mitochondrial pathways were documented including changes in mitochondrial respiratory function, metabolites and coenzymes, there was a lack of consistency across the studies, including results and study designs [9–28]. Furthermore, with the low numbers of studies in this field it is difficult to derive appropriate conclusions for mitochondrial dysfunction for the pathomechanism of ME/CFS [9–28].

Possible mechanisms of mitochondrial involvement in ME/CFS have been described in literature [9–28]. The presentation and severity of ME/CFS symptoms vary from patient to patient [29]. Due to the heterogenous nature of the illness it has been proposed that multiple pathological pathways are implicated in this condition including: muscle function, metabolism, mitochondria, neurological, cardiovascular and immunity [29]. Issues in fatty acid and amino acid metabolism as well as inefficient ATP synthesis have been suggested to have developed in ME/CFS patients due to dysregulation of mitochondrial or metabolic pathways [29].

ME/CFS patients have used a variety of different mitochondrial- based nutraceuticals including nicotinamide adenine dinucleotide hydrogen (NADH), coenzyme Q_10_ (CoQ_10_) and Acetyl L-Carnitine (ALC) as part of their treatment regime [2, 30–32]. Patients use these treatments either in isolation or in combination with a cocktail of other nutraceutical and/ or pharmaceutical- based products in the attempt to ameliorate their symptoms. A systematic appraisal of these reports and the impact they have on mitochondrial pathways affected in ME/CFS has not been conducted.

Using our previous publication as a basis we have expanded the search to assess the effectiveness of nutraceutical interventions in treating ME/CFS symptoms by measuring patient-centred outcomes such as fatigue levels [28].

## Methods

**Fig. 1:**
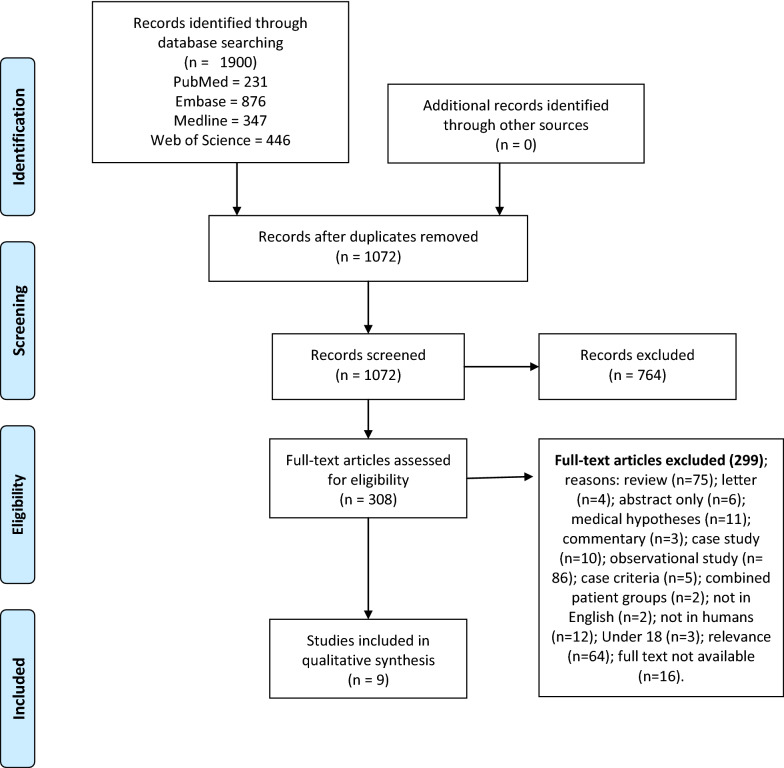
PRISMA flow diagram of literature search for included studies in this review of mitochondria interventions and ME/CFS

This systematic review followed Preferred Reporting Items for Systematic Reviews and Meta-Analyses (PRISMA) and Cochrane review guidelines (Fig. 1). Systematic searches of the databases Pubmed, Embase, Medline (EBSCO host) and Web of Science (via Clarivate Analytics) were conducted to collect relevant literature. Independent literature searches were conducted by RM and CB between January 1995 to 10th November 2020. No additional papers were found following reference list checking and citation searching. All potentially relevant papers have been included in this review (Additional file 1).

### Inclusion and exclusion criteria

Studies were selected if they contained at least one mitochondrial or intervention search term AND at least one ME/CFS search term (Additional file 1) as well as satisfying the following inclusion criteria: (i) interventional study published after 1994 (ii) study conducted on human participants aged 18 years or older; (iii) full-text available in English (iv) reporting of original research only; (v) diagnosis of ME/CFS was made using: FC (1994), CCC (2003), ICC (2011) or IOMC (2015) only; (vi) studies using nutraceuticals to target mitochondrial disruption in ME/CFS.

Studies were excluded from the review if they did not contain at least one mitochondrial or intervention search term AND at least one ME/CFS search term in the title or abstract. Articles were also excluded if they met any of the following criteria: (i) was an observational study (ii) written prior to 1995, as the study defining FC was published in December 15th 1994; (iii) not available as full- text; (iv) not published in English; (v) was a non-original study type including: reviews, duplicate studies or case reports; (vi) use of diagnostic criteria other than FC, CCC, ICC or IOMC; (vii) studies not within the scope of this review (Additional file 2).

### Selection of studies

All articles that were retrieved from each database were stored in the reference management software package Endnote X9.2. Duplicates were automatically removed and then the result was manually checked to confirm the removal of all duplicates. Titles and abstracts were screened for the listed key words. Articles that contained one mitochondrial or intervention term and one ME/CFS term were retained. Articles that did not contain the aforementioned key words were omitted. The remaining articles were assessed for suitability against inclusion and exclusion criteria and those that met eligibility criteria were selected. The described processes were conducted independently by authors RM and CB. The outcome of this screening process was discussed and deemed correct by both authors. The included articles were reassessed by all other listed authors for appropriateness.

### Data extraction

Relevant data were extracted from each of the studies (Table 1), this includes: (i) study design; (ii) treatment; (iii) reported adverse effects; (iv) treatment duration; (v) ME/CFS case definition; (vi) country; (vii) sample size; (viii) age of participants; (ix) sex, percentage of female participants; (x) illness duration; (xi) body mass index; (xii) weight; (xiii) duration of treatment; (xiv) washout period; (xv) treatment intervention; (xvi) control intervention; (xvii) fatigue outcome; (xviii) fatigue result.

**Table 1:** Summary of study and patient characteristics

References	Year	Study design	Treatment	Reported adverse effects	Treatment duration	Dx	Sample (n)		Age (years), mean (SD)		Sex, female %		Illness duration, Mean (SD)		BMI (kg m^−2^), mean (SD)		Weight (kg), mean (SD)	
							Tx	Con	Tx	Con	Tx	Con	Tx	Con	Tx	Con	Tx	Con
Castro-Marrero et al	2015	RCT, PAR	Coenzyme Q10 plus NADH	No reported adverse effects	8- weeks	Fukuda criteria	39	34	NR	NR	NR	NR	15.4 (8.9)	14.7 (6.2)	26.7 (5.2)	25.9 (2.4)	68.5 (14.6)	72.1 (13.7)
Castro-Marrero et al	2016	RCT, PAR	Coenzyme Q10 plus NADH	No reported adverse effects	8- weeks	Fukuda criteria	39	34	49.3 (7.1)	49.1 (8.4)	100%	100%	15.4 (8.9)	14.7 (6.2)	23.7 (5.2)	22.3 (2.4)	68.5 (14.6)	72.1 (13.7)
Forsyth et al	1999	RCP, CO	NADH	overly stimulated, mild loss of appetite, heartburn, increased incidence of gas and an odd taste and dryness in mouth	4-weeks	Fukuda criteria	26	NA	39.6	NA	65%	NA	7.2	NA	NR	NA	NR	NA
Fukuda et al	2016	RCT, PAR	Ubiquinol-10	No serious adverse effects	8-weeks	Fukuda criteria	17	14	34.8 (9.36)	39.5 (8.50)	76%	86%	NR	NR	NR	NR	NR	NR
		OPT	Ubiquinol-10	No serious adverse effects	12-weeks	Fukuda criteria	20	NA	36.8 (6.88)	NA	75	NA	10.25 (5.35)	NA	NR	NA	NR	NA
Kaiser et al	2015	POC	KPAX002	Dry mouth	12-weeks	Fukuda criteria	15	NA	45.4	NA	53.3%	NA	12.4	NA	NR	NA	NR	NA
Menon et al	2017	OPT	nutraceutical combination	No serious adverse effects	16-weeks	Fukuda criteria	10	NA	36.3 (10.46)	NA	70%	NA	11 (7.04)	NA	NR	NA	NR	NA
Montoya et al	2018	RCT, PAR	KPAX002	No significant adverse effects	12-weeks	Fukuda criteria	67	68	42.8	42.3	77.8%	66.2%	11.3	11.8	NR	NR	NR	NR
Ostojic et al	2016	RCT, CO	Guanidinoacetic Acid	No reported adverse effects	12- weeks	Fukuda criteria	21	NA	39.3 (8.8)	NA	100%	NA	NR	NA	NR	NA	62.8 (8.5)	NA
Vermeulen et al	2004	OPT	ALC and PLC	overstimulation and sleeplessness	24-weeks	Fukuda criteria	ALC: 30 PLC: 30 ALC + PLC: 30	NA	ALC: 37 (11) PLC: 38 (11) ALC + PLC: 42 (12)	NA	ALC: 76.7% PLC: 76.7% ALC + PLC: 76.7%	NA	ALC: 5.5 (1.0–23.0)^a^ PLC: 3.0 (0.5–25.0)^a^ ALC + PLC: 6.0 (1.0–21.0)^a^	NA	NR	NA	NR	NA

^a^Median (range)

ALC, Acetyl-l-Carnitine; BMI, Body Mass Index; CO, Cross-over design; Con, Control; NA, Not Applicable; NR, Not Recorded; OPT, Open labelled pilot trial; PLC, Propionyl-l-Carnitine; POC, Proof of Concept; RCT, Randomized Control Trial; SD, Standard Deviation; Tx, treatment

### Quality assessment

Quality and bias were assessed using the Rosendal scale [33]. This scale combines the PEDro scale [34], Jadad scoring system [35] and Delphi list [36]. This Rosendal scale was selected as the PEDro scale, Jadad scoring system and Delphi list have been extensively evaluated and validated. A Rosendal score of 60% is considered as excellent methodological quality [33]. Item 16 was excluded due to exercise performance outcomes not being measured in this review and item 15 was replaced with a checklist item that addresses a washout period in cross-over studies as conducted in Campagnolo et al.*’s* study [5]. No studies were excluded based on quality assessment results. Quality assessment was individually conducted by RM and CB.

## Results

A total of 1900 papers were retrieved from PubMed (231), Embase (876), Medline (347) and Web of Science (446). Duplicates were then removed, and inclusion and exclusion criteria were applied to the remaining articles. From this process the total number of articles was refined to nine. This selection process conducted according to PRISMA guidelines is summarised in Fig. 1.

### Overview of papers

Study characteristics are presented in Table 1. All included papers are intervention based. One study comprised of two experiments the initial experiment was an open-labelled pilot trial (OPT) and the subsequent experiment was a randomised control trial (RCT) with parallel design [37]. Four of the other included studies were RCT where two were crossover design and two were a parallel design [30, 31, 38, 39]. Two of the studies were OPT [40, 41]. One of the studies was a randomised control pilot trial [32]. The final study was a proof of concept study [42].

### Participant and study characteristics

Participant characteristics are also presented in Table 1. All nine papers used the FC to diagnose ME/CFS [30–32, 37–42]. The average sample size across the studies was 28.7 for treatment groups and 37.5 for the placebo control groups. The average age was 40.12 for treatment groups and 43.63 for the placebo control group. Females made up a greater proportion of the participants where 75.2% of the participants in the treatment group and 84% of the participants in the placebo control group were female. The average illness duration was 9.7 years for treatment groups and 13.7 years for the placebo control groups. All studies except one reported fatigue as a primary outcome [31, 32, 37–42]. To measure fatigue a variety of different resources were used including the: Fatigue Index Symptom Questionnaire (Fis-40), 50 item questionnaire based on the FC, Chalder Fatigue Score, visual analogue scale (VAS) for Fatigue and Multidimensional Fatigue Inventory (MFI) [31, 32, 37–42].

### Interventions on primary outcomes

Fatigue was the primary outcome measure in eight out of nine studies [31, 32, 37–42]. From these studies, six  found significant differences in fatigue levels following an intervention [31, 32, 39–42]. When receiving a combination treatment of CoQ_10_ and NADH a significant reduction in Fis-40 scores were reported in one study [31]. Additionally, consumption of NADH alone, also resulted in significantly reduced fatigue scores compared to the placebo control group [32]. Combination treatments including: KPAX002 (methylphenidate hydrochloride accompanied by a mitochondrial modulator) and another range of mitochondrial targeting nutrients significantly reduced symptom scores [39, 41, 42]. One KPAX002 study found that the most significant response was with those who had higher symptom severity. ALC and propionyl-l-carnitine (PLC) significantly reduced mental fatigue and general fatigue, respectively. However, combination treatments of ALC AND PLC treatments was a less effective intervention [40]. Two studies reported no significant changes in fatigue levels following intervention [37, 38]. One study’s primary endpoint was to investigate the effect of CoQ_10_ plus NADH supplementation on age-predicted maximum heart rate when completing a cycle ergometer task [30]. Participants in the intervention group reported significantly lower max HR. Perception of fatigue in this group was significantly reduced in all follow up visits in the intervention group compared with the placebo group [30].

### Interventions on secondary outcomes

Multiple secondary outcomes were investigated including physical or biochemical parameters, psychological, quality of life and pain scores. One study reported significantly higher nicotinamide adenine dinucleotide (NAD^+^/NADH), CoQ_10_, adenosine triphosphate (ATP) and citrate synthase levels as well as a significant reduction in lipoperoxides in ME/CFS patients using CoQ_10_ and NADH [31]. Another study found that patients using guanidinoacetic acid (GAA) had significantly higher muscular creatine levels as well as improved muscular strength and aerobic power [38]. In a study that investigated depression scores following intervention with ubiquinol-10, scores were found to be dependent on an increase in total plasma CoQ_10_ levels [37]. A study that investigated the effect of CoQ_10_ and NADH on max heart rate when completing a cycle ergometer task found no significant difference in sleep and pain scores, however, fatigue scores were significantly lower [30]. Vermeulen et al. investigated the effects of ALC, PLC and a combinatorial treatment of ALC and PLC [40]. Performance on the Stroop attention concentration test was significantly improved in all groups while pain scores were not significantly different between either of the groups [40].

### Quality assessment

Quality assessment scores for each study can be found in Additional file 2. Seven of the studies in this review were determined high quality (Rosendal score > 60%) [30–32, 37–40]. The most effectively addressed items were number one, six and ten. These items assess whether the study had a clear description of the inclusion and exclusion criteria, incorporation of baseline variables and whether details of participants who did not complete the study course were provided, respectively. The least addressed item was number nine which assesses the method of blinding participants and whether the effectiveness of blinding was described (Table 2).

**Table 2:** Summary of primary/secondary outcome results

References (date)	Primary/ Secondary outcome measures (s)	Results
Castro Marrero et al. (2015)	NAD + /NADH CoQ10 ATP Citrate Synthase Lipoperoxides	NAD + /NADH: significantly higher (p < 0.001) CoQ_10_: significantly higher (p < 0.05) ATP: significantly higher (p < 0.05) Citrate Synthase: significantly higher (p < 0.05) Lipoperoxides: significantly lower (p < 0.05)
Castro Marrero et al. (2016)	FIS-40	Sleep: NS Pain: NS Fatigue: significantly lower (p = 0.03)
Forsyth et al. (1999)	NA	NA
Fukuda et al. *(*2016)	CES-D	NS. Increase in depressive symptom scores were dependent on increase in total plasma CoQ_10_ levels
Kaiser et al. (2015)	NA	NA
Menon et al. (2017)	Clinical Global Impression Scale (CGI) Patient Global Impression Measures (PGI) Insomnia Severity Index (ISI)	CGI: Significantly improved (p = 0.014) PGI: NS ISI: Significantly improved (p = 0.017)
Montoya et al. (2018)	VAS for fatigue Concentration disturbance symptoms	VAS for fatigue: NS Concentration disturbance symptoms: NS
Ostojic et al. (2016)	Muscular Creatine Levels Muscular Strength and Aerobic Power	Muscular creatine levels: Significantly higher (p < 0.01) Muscular Strength and Aerobic Power: (p < 0.05)
Vermeulen et al. (2004)	Stroop attention concentration test McGill Pain Questionnaire	*Stroop attention concentration test* ALC: p < 0.001 PLC: p = 0.011 ALC + PLC: p = 0.004 *McGill Pain Questionnaire* ALC: NS PLC: p = NS ALC + PLC: NS

ALC, Acetyl-l-Carnitine; ATP, Adenosine Triphosphate; CES-D, Center for Epidemiological Studies Depression CGI, Clinical Global Impression Scale; CoQ10, Coenzyme Q10; FIS-40, Fatigue Impact Scale; ISI, Insomnia Severity Index; NAD, Nicotinamide Adenine Dinucleotide; NADH, Nicotinamide Adenine Dinucleotide Hydrogen; NA, Not Applicable; PGI, Patient Global Impression Measures; PLC, Propionyl-l-Carnitine; NS, Non-significant; VAS, Visual Analogue Scale

## Discussion

The use of nutritional supplements has been reported by ME/CFS patients for management of their condition [30–32, 37–42]. This systematic review aims to investigate the possible effect of mitochondrial-modifying nutraceuticals on ME/CFS patient outcomes such as fatigue and quality of life, sleep, and cognitive ability.

Mitochondrial disruption in ME/CFS patients was investigated previously in a systematic review conducted by Holden et al.[28] that reported inconsistent evidence linking mitochondrial disruption and ME/CFS [16, 19, 21, 26, 27]. No significant differences in mitochondrial deoxyribonucleic acid were found, strongly suggesting that ME/CFS is not a primary mitochondrial disorder [24]. The publication by Holden et al. only included observational studies [9–28]. This present interventional systematic review was conducted to complement Holden et al.’s study by investigating whether mitochondrial disruption has a role in ME/CFS pathomechanism as well as patient outcomes, when treated with mitochondrial-targeting nutraceuticals. This manuscript is the first to investigate this topic.

Key terms were derived from Nicolson et al.‘s review which featured an extensive list of nutraceutical agents used to treat mitochondrial dysfunction [43]. Limitations on relying on this developed list is exclusion of potentially relevant articles based on keywords selected. The final list of included articles has been reviewed and all appropriate articles have been incorporated in the present review. Some studies that investigated mitochondrial-targeting nutraceuticals that were on Nicolson’s list were not included in the final review due to not meeting criteria, for example, Comhaire et al. [44]. The diagnostic criteria used in this study is unclear therefore the manuscript was not selected [44]. This study investigated use of the nutraceutical, sodium dichloroacetate for treatment of ME/CFS [44].

Majority of the participants were female (75.2% for treatment group and 84% for placebo controls) with an average age of 40.12 years for the treatment group and 43.63 for controls. This is consistent with literature stating that ME/CFS is mostly reported in females aging between 35 and 45 years [19, 45]. Age and sex-matching participants is essential in mitochondrial studies due to age-related mitochondrial decline and sex-associated differences, including uncoupled respiration, citrate synthase activity and ATP levels [46]. One study specifically recruited only females to increase sample homogeneity [38]. Only two studies reported data on race or ethnicity where most of the participants were Caucasian [32, 39]. Lack of inclusion of minority groups, however, will result in a sample that doesn’t appropriately represent the population [47].

Information on diet restrictions was provided in two studies [38, 39]. Due to possible food-drug and food-food interactions, consumption of certain foods may influence response to treatment, hence, a diet record is a necessary consideration for future studies [48].

All the studies included in this review have utilised the FC to diagnose ME/CFS patients [2]. While the FC is the most widely utilised, is considered too broad and represents a more heterogenous set of patients with overlap with other illnesses such as depression [3]. Use of the more refined definitions including the CCC, ICC and IOMC may improve the overall sensitivity of these experiments; which is an important consideration for future studies [1, 3, 4].

Across the studies, an extensive range of different study types were identified including pilot trials and RCT. All cross-over studies effectively implemented a wash-out period [32, 38]. Some studies lacked an established placebo group [41, 42]. The studies that did have a placebo group indicated that there was a noticeable effect of the placebo [32]. Implementation of a placebo group is important to account for the placebo effect contribution to the observed results especially where subjective measures are concerned. Overall improvement in study design in future studies is paramount including repeating of open pilot studies as a RCT.

A range of different criteria to measure fatigue were used across the studies including: chalder fatigue score, Fis-40, visual analogue scale for fatigue and MFI. Due to this variety, it is difficult to adequately compare each study. One study used their own developed criteria based on the FC [32]. Reproducibility was tested for this criteria; however, this impact was only observed in one study and lacks validation [32]. Therefore, use of standardised, validated tools as conducted by all the other included studies is recommended for future research. Fatigue was calculated based on self-report questionnaires, these reports are subject to bias including selective recall and social desirability [49].

Tolerance is an important consideration for long-term use of natural supplements. Tolerance was assessed in eight of the studies [30–32, 37–41]. Across the studies adverse effects that were described include over-stimulation, heartburn, dry mouth and mild loss of appetite [30–32, 37–41]. Due to concomitant medications not being controlled in the study, understanding the adverse effects caused by the nutraceuticals alone is hindered [41]. Majority of the studies investigated short-term side effects whereas long-term effects of supplement use for ME/CFS patients has not been determined. Replication of these studies for longer durations to assess long-term implications of use is necessary.

One possible outcome highlighted in our previous mitochondrial paper is that disruption of mitochondrial pathways may be one portion of a much more complex, multifactorial pathological process involving multiple pathways such as Ca^2+^ signalling [18, 19]. Some mitochondrial processes are Ca^2+^-dependent [7]. Additionally, mitochondria have a role in Ca^2+^-homeostasis [50]. Importantly, impairments in Ca^2+^-dependent pathways results in oxidative stress that have been reported in ME/CFS patients [32, 39–42].

A review by Wood et al. investigated mitochondrial involvement in ME/CFS, oxidative stress and therapeutic potential of antioxidant therapies including CoQ_10_ in ME/CFS patients [51]. There is still insufficient evidence of mitochondrial involvement in ME/CFS and further research is required to establish appropriate grounds for treatments [28].

Currently there is limited evidence available on the efficacy of mitochondria interventions for treatment of ME/CFS symptoms. Additional well-designed studies are required to understand the impact of these nutraceuticals on ME/CFS patients. Furthermore, in a letter to the editor written by Ostojic et al. the role of creatine in treatment of ME/CFS was discussed. The overall message of the excerpt was to refrain from use until better evidence is available including: dosing, treatment duration and side-effects [52]. This suggestion can be applied to all other treatments assessed in this systematic review [52].

### Quality assessment

A Rosendal score of 60% is considered as excellent methodological quality [33]. This score was achieved by seven of the included studies [30–32, 37–40]. All papers were effective at describing eligibility and exclusionary criteria. Majority of the studies received lower scores due to lack of randomisation and blinding of participants and assessors. The least addressed item was number nine. Item nine assesses the method of blinding and whether the successfulness of blinding has been discussed. These details were only provided by one study. Important considerations for future studies include selection and justification of an appropriate sample size, incorporation of randomisation and blinding processes and ensuring that evaluation of blinding measures have been discussed. Additionally, appropriate statistical measures must be used; this includes, selection of statistical tests based on normality assessment as well as adjusting for multiple parameters. Sample size must also be considered statistically sufficient.

## Conclusion

The aim of this systematic review was to evaluate available research on mitochondrial-based nutraceutical interventions. Due to numerous limitations associated with these studies such as low availability of studies investigating this topic, lack of consistency of measuring tools for fatigue used, low sample size and short study duration, it is difficult to make conclusions on whether these interventions have an effect on mitochondrial function in ME/CFS patients. Therefore, future well-designed studies are required to determine mitochondrial involvement in ME/CFS pathology and the possible effectiveness of these interventions in treating ME/CFS. These findings may help direct future research in this field.

## Supplementary Information

**Additional file 1.** Raw search code.

**Additional file 2.** Quality assessment summary and Rosendal score of studies KB).

## Data Availability

All data generated or analysed during this study are included in this published article.

## Abbreviations

ALC: Acetyl-l-Carnitine
ATP: Adenosine triphosphate
Ca2 +: Calcium
CCC: Canadian Consensus Criteria
CoQ_10_: Coenzyme Q_10_
ETC: Electron Transport Chain
HC: Healthy control
FIS: Fatigue Index Symptom
FC: Fukuda Criteria
GAA: Guanidinoacetic acid
ICC: International Consensus Criteria
IOMC: Institute of Medicine Criteria
MeSH: Medical subject headings
mRNA: Messenger Ribonucleic Acid
mtDNA: Mitochondrial DNA
MFI: Multidimensional Fatigue Inventory ME/CFS: Myalgic Encephalomyelitis/ Chronic Fatigue Syndrome
NK: Natural Killer
NAD: Nicotinamide adenine dinucleotide
NADH: Nicotinamide adenine dinucleotide hydrogen
OPT: Open-labelled pilot trial
PRISMA: Preferred Reporting Items for Systematic Reviews
PLC: Propionyl-l-Carnitine
RCT: Randomised control trials
VAS: Visual Analogue Scale
