# Supplementary material for: A systematic review of nutraceutical interventions for mitochondrial dysfunctions in myalgic encephalomyelitis/chronic fatigue syndrome
Source: J Transl Med. 2021 Feb 17;19:81. doi: 10.1186/s12967-021-02742-4 (PMC7890871; doi:10.1186/s12967-021-02742-4)
Supplement: Supplementary file 1 — Additional file 1. Raw search code. [file 12967_2021_2742_MOESM1_ESM.docx]

**Raw Search code**

Contains FIRST PASS + Vitamin C (Ascorbic Acid) + Vitamin D + Vitamin E + thiamine + riboflavin +Magnesium + Calcium + Phosphates +membrane phospholipids + unsaturated fatty acids +creatine + pyruvic Acid + Ubiquinone + Ubiquinol + Thioctic acid + NAD +Niacin + Carnitine + dichloroacetate + Curcumin + schisandrin

Pubmed

(("Fatigue Syndrome, Chronic"[Mesh])) AND (("Mitochondria" [Mesh]) OR ("Mitochondr*"[All Fields]) OR ("Energy Metabolism"[All Fields]) OR (“Q10”[All Fields]) OR (“Pyruvate Dehydrogenase”[All Fields]) OR (“Electron Transport Chain”[All Fields]) OR (“ATP”[All Fields]) OR (“ADP”[All Fields]) OR (“TCA Cycle”[All Fields]) OR (“Citric Acid Cycle”[All Fields]) OR ("Ascorbic Acid"[Mesh]) OR ("Vitamin D"[Mesh]) OR ("Vitamin E"[Mesh]) OR ("Thiamine"[Mesh]) OR ("Riboflavin"[Mesh]) OR ("Magnesium"[Mesh]) OR ("Calcium"[Mesh]) OR ("Phosphates"[Mesh]) OR ("Fatty Acids, Unsaturated"[Mesh]) OR ("Creatine"[Mesh]) OR ("Pyruvic Acid"[Mesh]) OR ("Ubiquinone"[Mesh]) OR ("Ubiquinol"[All Fields]) OR ("Thioctic Acid"[Mesh]) OR ("NAD"[Mesh]) OR ("Niacin"[Mesh]) OR ("Carnitine"[Mesh]) OR ("Curcumin"[Mesh]) OR ("Membrane Phospholipid*"[All Fields]) OR ("dichloroacetate"[All Fields]) OR (“Schisandrin"[All Fields]))

SCOPUS, MEDLINE AND EMBASE

(( "Chronic Fatigue Syndrome") OR ( "Myalgic Encephalomyelitis") OR (“Encephalomyelitis, Myalgic”) OR (“Chronic Fatigue Syndromes”) OR (“Fatigue Syndromes, Chronic”) OR (“Chronic Fatigue-Fibromyalgia Syndrome”) OR (“Chronic Fatigue Fibromyalgia Syndrome”) OR (“Chronic Fatigue Fibromyalgia Syndromes”) OR (“Fatigue-Fibromyalgia Syndrome, Chronic”) OR (“Fatigue-Fibromyalgia Syndromes, Chronic”) OR (“Postviral Fatigue Syndrome”) OR (“Infectious Mononucleosis-Like Syndrome, Chronic”) OR (“Infectious Mononucleosis Like Syndrome, Chronic”) OR (“Royal Free Disease”) OR (“Chronic Fatigue and Immune Dysfunction Syndrome”) OR (“Chronic Fatigue Disorder”) OR (“Chronic Fatigue Disorders”) OR (“Fatigue Disorder, Chronic”) OR (“Fatigue Disorders, Chronic”) OR ( "systemic exertion intolerance" ) OR (“Fatigue Syndrome, Postviral”) OR (“Fatigue Syndromes, Postviral”) OR (“Postviral Fatigue Syndromes”)) AND ((“Mitochondria”) OR (“Mitochondrion”) OR (“Mitochondrial Contraction”) OR (“Contraction, Mitochondrial”) OR (“Contractions, Mitochondrial”) OR (“Mitochondrial Contractions”) OR ("Mitochondri*") OR (“Energy Metabolism”) OR (“Q10”) OR (“Pyruvate dehydrogenase”) OR (“Electron transport chain”) OR (“ATP”) OR (“ADP”) OR (“TCA cycle”) OR (“Citric acid cycle”) OR (“Ascorbic Acid”) OR (“Acid, Ascorbic”) OR (“L-Ascorbic Acid”) OR (“Acid, L-Ascorbic”) OR (“L Ascorbic Acid”) OR (“Vitamin C”) OR (“Hybrin”) OR (“Magnorbin”) OR (“Sodium Ascorbate”) OR (“Ascorbate, Sodium”) OR (“Ascorbic Acid, Monosodium Salt”) OR (“Ferrous Ascorbate”) OR (“Ascorbate, Ferrous”) OR (“Magnesium Ascorbate”) OR (“Ascorbate, Magnesium”) OR (“Magnesium di-L-Ascorbate”) OR (“Magnesium di L Ascorbate”) OR (“di-L-Ascorbate, Magnesium”) OR (“Magnesium Ascorbicum”) OR (“Vitamin D”) OR (“Vitamin E”) OR (“Thiamine”) OR (“Thiamin”) OR (“Vitamin B1”) OR (“Aneurin”) OR (“Vitamin B 1”) OR (“Thiamine Mononitrate”) OR (“Mononitrate, Thiamine”) OR (“Riboflavin”) OR (“Vitamin G”) OR (“Vitamin B2”) OR (“Vitamin B 2”) OR (“Magnesium") OR (“Calcium”) OR (“Blood Coagulation Factor IV”) OR (“Factor IV, Coagulation”) OR (“Calcium-40”) OR (“Calcium 40”) OR (“Factor IV”) OR (“Phosphates”) OR (“Phosphates, Inorganic”) OR (“Phosphate”) OR (“Inorganic Phosphates”) OR (“Orthophosphate”) OR (“Fatty Acids, Unsaturated”) OR (“Acids, Unsaturated Fatty”) OR (“Unsaturated Fatty Acids”) OR (“Polyunsaturated Fatty Acids”) OR (“Acids, Polyunsaturated Fatty”) OR (“Fatty Acids, Polyunsaturated”) OR ("Creatine") OR (“Pyruvic Acid”) OR (“Acid, Pyruvic”) OR (“Pyruvate”) OR ("Ubiquinone") OR (“Coenzyme Q”) OR (“Ubiquinol”) OR (“Thioctic Acid”) OR (“alpha-Lipoic Acid”) OR (“Acid, alpha-Lipoic”) OR (“alpha Lipoic Acid”) OR (“Lipoic Acid”) OR (“Azulipont”) OR (“biomo-lipon”) OR (“biomo lipon”) OR (“biomolipon”) OR (“alpha-Liponsaure von ct”) OR (“alpha Liponsaure von ct”) OR (“alphaLiponsaure von ct”) OR (“espa-lipon”) OR (“espa lipon”) OR (“espalipon”) OR (“Pleomix-Alpha N”) OR (“Pleomix Alpha N”) OR (“PleomixAlpha N”) OR (“alpha-Liponaure Heumann”) OR (“alpha Liponaure Heumann”) OR (“alphaLiponaure Heumann”) OR (“Neurium”) OR (“Pleomix-Alpha”) OR (“Pleomix Alpha”) OR (“PleomixAlpha”) OR (“Juthiac”) OR (“Alphaflam”) OR (“duralipon”) OR (“MTW-Alphaliponsaure”) OR (“MTW Alphaliponsaure”) OR (“MTWAlphaliponsaure”) OR (“Fenint”) OR (“Liponsaure-ratiopharm”) OR (“Liponsaure ratiopharm”) OR (“Liponsaureratiopharm”) OR (“alpha-Vibolex”) OR (“alpha Vibolex”) OR (“alphaVibolex”) OR (“Alpha-Liponsaure Sofotec”) OR (“Alpha Liponsaure Sofotec”) OR (“AlphaLiponsaure Sofotec”) OR (“Alpha-Lipon Stada”) OR (“Alpha Lipon Stada”) OR (“AlphaLipon Stada”) OR (“Tromlipon”) OR (“Verla-Lipon”) OR (“Verla Lipon”) OR (“VerlaLipon”) OR (“Thioctacide T”) OR (“Thioctacid”) OR (“Alpha-Lipogamma”) OR (“Alpha Lipogamma”) OR (“AlphaLipogamma”) OR (“Thiogamma oral”) OR (“Thiogamma Injekt”) OR (“Injekt, Thiogamma”) OR (“Alpha-Lippon AL”) OR (“Alpha Lippon AL”) OR (“AlphaLippon AL”) OR (“NAD") OR (“Dihydronicotinamide Adenine Dinucleotide”) OR (“Adenine Dinucleotide, Dihydronicotinamide”) OR (“Dinucleotide, Dihydronicotinamide Adenine”) OR (“Diphosphopyridine Nucleotide”) OR (“Nucleotide, Diphosphopyridine”) OR (“Nadide”) OR (“Nicotinamide-Adenine Dinucleotide”) OR (“Dinucleotide, Nicotinamide-Adenine”) OR (“Nicotinamide Adenine Dinucleotide”) OR (“Coenzyme I”) OR (“DPN”) OR (“NADH”) OR (“Niacin”) OR (“Nicotinic Acid”) OR (“3-Pyridinecarboxylic Acid”) OR (“3 Pyridinecarboxylic Acid”) OR (“Niacin Lithium Salt, Hemihydrate”) OR (“Niacin Potassium Salt”) OR (“Potassium Salt, Niacin”) OR (“Niacin Sodium Salt”) OR (“Sodium Salt, Niacin”) OR (“Niacin Tartrate”) OR (“Tartrate, Niacin”) OR (“Niacin Copper (2+) Salt”) OR (“Niacin Hydrochloride”) OR (“Hydrochloride, Niacin”) OR (“Niacin Iron (2+) Salt”) OR (“Niacin Tosylate”) OR (“Tosylate, Niacin”) OR (“Niacin Zinc Salt”) OR (“Nicamin”) OR (“Nico-400”) OR (“Nico 400”) OR (“Nico400”) OR (“Nicobid”) OR (“Nicocap”) OR (“Nicolar”) OR (“Nicotinate”) OR (“Wampocap”) OR (“Enduracin”) OR (“Induracin”) OR (“Niacin Calcium Salt”) OR (“Lithium Nicotinate”) OR (“Nicotinate, Lithium”) OR (“Niacin Aluminum Salt”) OR (“Aluminum Salt, Niacin”) OR (“Niacin Ammonium Salt”) OR (“Niacin Lithium Salt”) OR (“Niacin Cobalt (2+) Salt”) OR (“Niacin Magnesium Salt”) OR (“Niacin Manganese (2+) Salt”) OR ("Carnitine") OR (“Levocarnitine”) OR (“Vitamin BT”) OR (“L-Carnitine”) OR (“L Carnitine”) OR (“Bicarnesine”) OR (“Curcumin”) OR (“Turmeric Yellow”) OR (“Yellow, Turmeric”) OR (“Diferuloylmethane”) OR (“Membrane Phospholipid”) OR (“dichloroacetate”) OR (“Schisandrin”))
