# Supplementary material for: A systematic review of nutraceutical interventions for mitochondrial dysfunctions in myalgic encephalomyelitis/chronic fatigue syndrome
Source: J Transl Med. 2021 Feb 17;19:81. doi: 10.1186/s12967-021-02742-4 (PMC7890871; doi:10.1186/s12967-021-02742-4)
Supplement: Supplementary file 2 — Additional file 2. Quality assessment summary and Rosendal score of studies KB). [file 12967_2021_2742_MOESM2_ESM.docx]

Quality assessment summary and Rosendal score of studies

| **Reference** | **Eligibility^1^** | **Randomisation^2^** | **Method of Randomisation^3^** | **Sample size justified^4^** | **Pre-trial conditions^5^** | **Baseline measures^6^** | **Blinding of subjects^7^** | **Blinding of investigators^8^** | **Method and Evaluation of Blinding^9^** | **Non Completers Described^10^** | **Statistics described^11^** | **Measures and Variability Described^12^** | **Between Group Stats Comparisons^13^** | **Method used to access adverse effects was described^14^** | **Appropriate washout period^15^** | **% Score^** |
| --- | --- | --- | --- | --- | --- | --- | --- | --- | --- | --- | --- | --- | --- | --- | --- | --- |
| Castro-Marrero *et al* (2015) | 1 | 1 | 0 | 0 | 1 | 1 | 1 | 1 | 0 | 1 | 1 | 1 | 1 | 1 | - | **78.6%** |
| Castro-Marrero *et al* (2016) | 1 | 1 | 1 | 0 | 1 | 1 | 1 | 1 | 0 | 1 | 1 | 1 | 1 | 1 | - | **85.7%** |
| Forsyth *et al* (1999) | 1 | 1 | 0 | 0 | 0 | 1 | 1 | 1 | 0 | 1 | 1 | 0 | 0 | 1 | 1 | **60.0%** |
| Fukuda *et al* (2016) | 1 | 1 | 1 | 1 | 1 | 1 | 1 | 1 | 0 | 1 | 1 | 1 | 1 | 0 | - | **85.7%** |
| Kaiser *et al* (2015) | 1 | 0 | 0 | 0 | 1 | 1 | 0 | 0 | 0 | 1 | 1 | 1 | - | 0 | - | **46.2%** |
| Menon *et al* (2017) | 1 | 0 | 0 | 0 | 0 | 1 | 0 | 0 | 0 | 1 | 1 | 1 | - | 0 | - | **46.2%** |
| Montoya *et al* (2018) | 1 | 1 | 1 | 0 | 1 | 1 | 1 | 1 | 0 | 1 | 0 | 1 | 1 | 1 | - | **78.6%** |
| Ostojic *et al* (2016) | 1 | 1 | 1 | 1 | 1 | 1 | 1 | 1 | 0 | 1 | 1 | 1 | 1 | 1 | 1 | **93.3%** |
| Vermeulen *et al* (2004) | 1 | 1 | 1 | 1 | 0 | 1 | 0 | 0 | 0 | 1 | 1 | 1 | 1 | 0 | - | **64.3%** |
|  |  |  |  |  |  |  |  |  |  |  |  |  |  |  |  |  |
|  |  |  |  |  |  |  |  |  |  |  |  |  |  |  |  |  |

^1^ A clear description of the inclusion and exclusion criteria

^2^ The trial was randomised

^3^ The method used to generate the random allocation sequence, including details of any restrictions (e.g. blocking, stratification) was described

^4^Sample size was justified (e.g. power calculation)

^5^Attempts were made to control and/or monitor pre-trial conditions (e.g. diet, exercise, nutritional status)

^6^Design incorporated measures of important baseline variables (e.g. fatigue, quality of life, physical activity and/or psychological wellbeing)

^7^There was blinding of all subjects

^8^There was blinding of all investigators involved in the trials

^9^Both the method of blinding and the evaluation of the successfulness of blinding were described

^10^Details were provided regarding the inability of a subject to complete study requirements

^11^Statistical methods used to compare groups for primary outcome measure(s)*, and methods for additional analyses, such as sub group analyses and adjusted analyses, were described

^12^Both point measures and measures of variability for the primary outcome measure(s) were provided^

^13^The results of between group statistical comparison were reported for the primary outcome measure(s)* (e.g. an estimated effect size), and its precision (e.g. 95% CI)

^14^The method used to assess adverse effects were described

^15^If an appropriate washout periods was used for crossover trials

*If primary outcome measure not stated then key measure used

^Scoring: the final percentage score is determined by dividing the number of 'yes' scores (yes=1, No = 0) by the total number of applicable items (i.e. 16 minus NA (-) items)

**Castro – Marrero *et al,* 2015**

1. Inclusion criteria include ME/CFS patients that meet the Fukuda criteria. Exclusion criteria include: acute infectious diseases in the previous 4- weeks, past or present neurological, psychiatry (depression/anxiety), metabolic, autoimmune, allergy-related diseases, dermal or chronic inflammation, smoking, alcohol abuse, oral diseases and those who are medicated with glucocorticoids, statins or antidepressant/ anxiolytic drugs.
2. The trial was randomised.
3. Patients were randomized in a double-blind fashion, 1:1 ratio.
4. Sample size was not justified.
5. All patients had a sedentary lifestyle. Levels of NADH and COQ10 in blood cells was quantified prior to- and post-treatment.
6. Yes, fatigue was an important baseline measurement used (the FIS scale).
7. Yes, it was a double-blind study.
8. Yes, it was a double-blind study.
9. The method of blinding was determined but the successfulness of this process was not described.
10. 33 participants were excluded on the grounds of not meeting inclusion criteria. No other participant was excluded for other reasons.
11. Yes, statistical data was provided. To compare CFS patients treated with CoQ10 plus NADH supplementation or placebo between post-treatment vs. baseline, a two-way variance (ANOVA) analysis.
12. Point measures and measures of variability are provided.
13. Yes, the between group comparison was provided.
14. Yes, adverse effects were recorded at each of the study visits and defined according to Medical Dictionary for regulatory activities terminology.
15. N/A

**Castro – Marrero *et al,* 2016**

1. Inclusion criteria include ME/CFS patients that meet the Fukuda criteria. Exclusion criteria include: acute infectious diseases in the previous 4- weeks, past or present neurological, psychiatry (depression/anxiety), metabolic, autoimmune, allergy-related diseases, dermal or chronic inflammation, smoking, alcohol abuse, oral diseases and those who are medicated with glucocorticoids, statins or antidepressant/ anxiolytic drugs.
2. The trial was randomised.
3. Patients were randomized in a double-blind fashion, 1:1 ratio – computer generated list using STATA 9.0.
4. Sample size was not justified.
5. All patients had a sedentary lifestyle. Levels of NADH and COQ10 in blood cells was quantified prior to- and post-treatment.
6. Yes, fatigue was an important baseline measurement used (the FIS scale).
7. Yes, it was a double-blind study.
8. Yes, it was a double-blind study.
9. The method of blinding was determined but the successfulness of this process was not described.
10. 33 participants were excluded on the grounds of not meeting inclusion criteria. No other participant was excluded for other reasons.
11. Yes, statistical data was provided. To compare CFS patients treated with CoQ10 plus NADH supplementation or placebo between post-treatment vs. baseline, a two-way variance (ANOVA) analysis.
12. Point measures and measures of variability are provided.
13. Yes, the between group comparison was provided.
14. Yes, adverse effects were recorded at each of the study visits and defined according to Medical Dictionary for regulatory activities terminology.
15. N/A

**Forsyth et al (1999)**

1. Inclusion criteria included participants who fulfilled the Fukuda definition criteria for ME/CFS/SEID. Those who took medications were able to participate but they were listed as concomitant medications. Exclusionary criteria included patient groups that could be explained by another medical or psychiatric illness. Those with substance abuse or with pre-existing illnesses that may influence fatigue levels such as hyperthyroidism, cancer, AIDS, severe anemia etc were also excluded. Additional exclusionary criterion includes participants who took antidepressants, lithium, neuroleptics and monoamine inhibitors.
2. The trial was randomised.
3. Participants were randomly allocated, however, the process by which this occurred was not described.
4. Sample size was not justified.
5. Attempts were not made to monitor or control pretrial conditions.
6. Yes, baseline fatigue was measured using 50 item questionnaire based on CDC criteria for ME/CFS.
7. Yes, it was a double-blind study
8. Yes, it was a double-blind study
9. The method of blinding was not described. The successfulness of the process was determined using a self-evaluated questionnaire.
10. Two participants were withdrawn from the study due to non-compliance and nine left due to use of psychotropic drugs.
11. Repeated measures analysis was conducted. Data was analysed using a test of difference between two proportions.
12. Point measures were provided but measures of variability were not provided.
13. Between group analysis were not provided.
14. Subjects were monitored for toxicity through appropriate laboratory testing.
15. Yes the washout period was 4 weeks.

**Fukuda et al ()**

1. Inclusion criteria was patients diagnosed with Fukuda criteria. Exclusion criteria was also provided: alternative diseases that induce fatigue, pregnancy or lactation, allergies to certain materials.
2. Yes the study was randomized but there was a portion of an open label study included in this study.
3. Study was randomised using block stratification (1:1)
4. Target results were observed in an open label study with 20 participants therefore 20 participants were also used in the RCT.
5. Those who were previously taking Ubiquinol were included after a wash out period of 4 weeks. There was no significant difference in baseline levels of plasma CoQ10 levels.
6. Baseline fatigue was measured using Chalder Fatigue Scale.
7. Yes it was a double blind study
8. Yes it was a double blind study
9. The original code number was placed in a sealed envelope. Successfulness of blinding was not described.
10. One participant discontinued due to deconditioning, another dropped out due to incidence of diarrhea before the supplementation, 5 patients withdrew their consent.
11. A T test was used. Chi square was used for categorical data. Correlation between the changes in total CoQ10 concentrations and each parameter each supplementation group was tested using Spearman rank-sum test.
12. Point measures and measures of variability are provided.
13. Yes between group statistical analysis was provided: One way analysis of variance.
14. Subjects were monitored for toxicity. Methods weren’t described.
15. N/A

**Kaiser et al (2015)**

1. Inclusion criteria is patients being diagnosed with the Fukuda criteria and had a normal safety laboratory profile. Participants were excluded if they have any additional conditions that may contribute to their fatigue including systemic treatment for cancer (within the past two years), major depressive disorder, diabetes mellitus and fibromyalgia. Participants were also excluded if they were pregnant, were currently taking prescription medication.
2. No, this study is an open label pilot trial.
3. As above – no randomisation.
4. Sample is a pilot trial used to determine sample sizes – justification for sample size was not provided.
5. Patients were requested to maintain fluid intake to 6-8 glasses per day and not substantially increase their level of activity.
6. Baseline fatigue was measured using CIS and VAS checklists.
7. No, they were not blinded.
8. No, they were not blinded.
9. Experiment was not blinded.
10. One subject withdrew consent after the 4-week study visit.
11. Total CIS score, CIS concentration disturbances subscore and the VAS for fatigue and concentration disturbances as 12 weeks were compared to baseline values with a paired T-test.
12. Point measures and measures of variability are provided.
13. N/A
14. No method to assess adverse effects was described.
15. N/A

**Menon et al (2017)**

1. Inclusion criteria include both males and females aged 18-65, fulfil the 1994 Fukuda guidelines for ME/CFS/SEID, are able to consent to participation, stable treatment. Exclusion criteria include: known or suspected active and unstable systemic medical disorder, individuals who had a major depressive episode in the past two years prior to diagnosis of ME/CFS, acute suicidality, individuals with a current diagnosis of a psychological disorder, gastrointestinal ulcers or renal stones, those who are pregnant or have a diagnosis of epilepsy, those who are currently taking study preparations (2 week washout period was required for those taking that wanted to participate).
2. Study was not randomised – open labelled pilot study.
3. As above – not randomised.
4. Sample size was determined based on convenience.
5. Attempts were not made to monitor or control pretrial conditions.
6. Baseline fatigue was measured using the Chalder Fatigue scale.
7. No, they were not blinded.
8. No, they were not blinded.
9. Experiment was not blinded.
10. Two participants withdrew from the study at week four and week twelve, both due to work commitments.
11. Repeated measures linear mixed effects models were used to test the effect of the intervention on primary and secondary outcomes.
12. Point measures and measures of variability are provided.
13. N/A
14. Adverse effects were monitored throughout the study, however, the method used was not described.
15. N/A

**Montoya et al (2018)**

1. Inclusion criteria includes: participants who are between 18-59 and met the 1994 Fukuda criteria. Exclusion criteria includes: those who are pregnant, active substance abuse, major depression, active medical conditions where treatment with methylphenidate hydrocholoride may be contraindicated, use of anxiety medications, use of medications such as monoamine oxidase inhibitors, other CNS stimulants and narcotic opioids.
2. Yes study was randomized.
3. Randomization codes were automatically provided by the electronic research management system used in this trial. Randomization allocation was 1:1 to active treatment and placebo, respectively.
4. Decisions regarding sample size was not discussed.
5. Participants were off any nutritional, herbal or caffeine containing – supplements or any pseudoephedrine containing products throughout the duration of the trial.
6. Yes, the checklist individual strength (CIS) questionnaire was used.
7. Yes, this was a double- blind study.
8. Yes, this was a double- blind study.
9. Details on blinding was not provided.
10. 11 participants had adverse effects from the treatment leading to early withdrawal from the study.
11. Comparison of mean change from baseline in treatment and placebo groups for the primary endpoint: the CIS score. No description of statistical tests used have been provided.
12. Point measures and measures of variability are provided.
13. CIS total scores were analysed with a repeated-measures mixed effects model.
14. Safety was assessed using summaries of adverse events for each treatment group and visit.
15. N/A

**Ostojic et al (2016)**

1. Inclusion criteria includes participants who were 18 and over and fulfilled the 1994 Fukuda definition. Exclusion criteria included: psychiatric comorbidity, use of any dietary supplement within 4 weeks of the study period, unwillingness to attend follow-up analysis and pregnancy.
2. Yes, study was randomised.
3. Randomisation was computer generated.
4. Sample size was in accordance of the power analysis.
5. Exclusive recruitment of women to increase sample homogeneity occurred.
6. Yes, the multidimensional fatigue inventory was used as a baseline measure
7. Yes, this was a double- blind study.
8. Yes, this was a double- blind study.
9. Details on blinding was not provided.
10. Seven participants were lost during the intervention period – reasons were not connected to the study.
11. Two-way mixed model ANOVA with repeated measures to include changes that existed between participant responses over time was used.
12. Point measures and measures of variability are provided.
13. Yes – refer to 11
14. Adverse effects were monitored through an open-ended questionnaire
15. There was a two-month wash out period

**Vermeulen et al (2004)**

1. Exclusion criteria was patients with an underlying cause for Fatigue, substance misuse, severe psychiatric disorder. Inclusion criteria include patients diagnosed with the 1994 Fukuda criteria.
2. Yes, study was randomised.
3. Randomisation occurred by using sealed envelopes and blocks of six patients (gender stratified)
4. Yes, sample size was based on two preliminary studies.
5. Attempts were not made to monitor or control pretrial conditions.
6. Yes, Clinical Global Impression of Change was used.
7. No, it was an open labelled study
8. No, it was an open labelled study.
9. It was an open labelled study.
10. Eight patients dropped out due to side effects.
11. Pearson’s correlation test for continuous data and Spearman rank correlation test for non-normal data.
12. Point measures and measures of variability are provided.
13. Changes during therapy were tested by Friedman’s test for multiple related variables.
14. Adverse effects were not monitored throughout the study or the method used was not described.
15. N/A
